# Memorials to John Snow – Pioneer in anaesthesia and epidemiology

**DOI:** 10.1177/09677720211013807

**Published:** 2021-05-07

**Authors:** Neil G Snowise

**Affiliations:** 1 Institute of Pharmaceutical Science, Faculty of Life Sciences and Medicine, 405987 King's College, London, UK

**Keywords:** Public health, memorials, epidemiology, anaesthesia, legacy

## Abstract

John Snow was an English physician and a founding father of epidemiology, whose name is inextricably linked with tracing the source of the 1854 cholera outbreak in Soho, which killed over 600 people. Despite his recommendation to remove the water pump handle and thus reduce the spread of cholera, his theory of faecal–oral transmission was not widely believed until after his death. Furthermore, he also pioneered substantial achievements in the development of anaesthesia. He studied both chloroform and ether, improving the accuracy of their delivery. In his obstetric practice, he achieved the feat of obtaining satisfactory analgesia with a safer technique and is remembered for administering chloroform to Queen Victoria, during the delivery of her last two children. There are several interesting and unusual memorials to Snow, ranging from replica water pumps, blue plaques and a public house named after him. The most recent new memorial was erected in 2017, in his home town of York, which commemorates his origins and his subsequent contribution to curbing the cholera outbreak. All the memorials commemorate his achievements, which remain relevant today. Public health and epidemiology expertise is required in the current world of the COVID-19 pandemic, where his legacy remains as important as ever.

John Snow (1813–1858) was an English physician, a leader in the development of anaesthesia and a founding father of epidemiology.^1–3^ His name is inextricably linked with tracing the source of the infamous 1854 cholera outbreak in Soho, London, which killed over 600 people. He was equally famed as an anaesthetist, studying both chloroform and ether. Queen Victoria asked Snow to administer chloroform to her during the delivery of her last two children.

There are many famous doctors who have memorials dedicated to their achievements, but most take the form of a wall plaque, bust or statue. As well as plaques, Snow appears to be unique in having both a pub named after him and two replicas of the infamous water pump. Furthermore, his life and works are commemorated by the John Snow Society in London^4^ and by a dedicated website at the University of California, Los Angeles, Department of Epidemiology.^5^ It seems fitting to remember him during this COVID-19 pandemic, when public health and epidemiology remain as crucial and important as ever, in our fight against disease. Science has identified the route of COVID transmission, whereas, in Snow's day, the transmission of cholera was hotly disputed. However, similarities remain, since both diseases are lethal, contact tracing is crucial and neither (at the time) had a curative treatment.

He was born in York in 1813, the first of nine children born to William and Frances Snow. He was born in their North Street home, which was then one of the poorest parts of York, from humble beginnings; it is here that the newest memorial to Snow lies, unveiled in March 2017 by York Civic Trust.^6,7^

The John Snow Memorial in York has at its centre a restored Victorian iron hand pump, with the pump handle removed, similar to ones he would have been familiar with. A new blue plaque to Snow and interpretation board with an overview of Snow's biography and the history of cholera accompany the water pump (Figure 1). The unveiling ceremony was attended by descendants of John Snow, including his great-great nephew.

**Figure 1.**
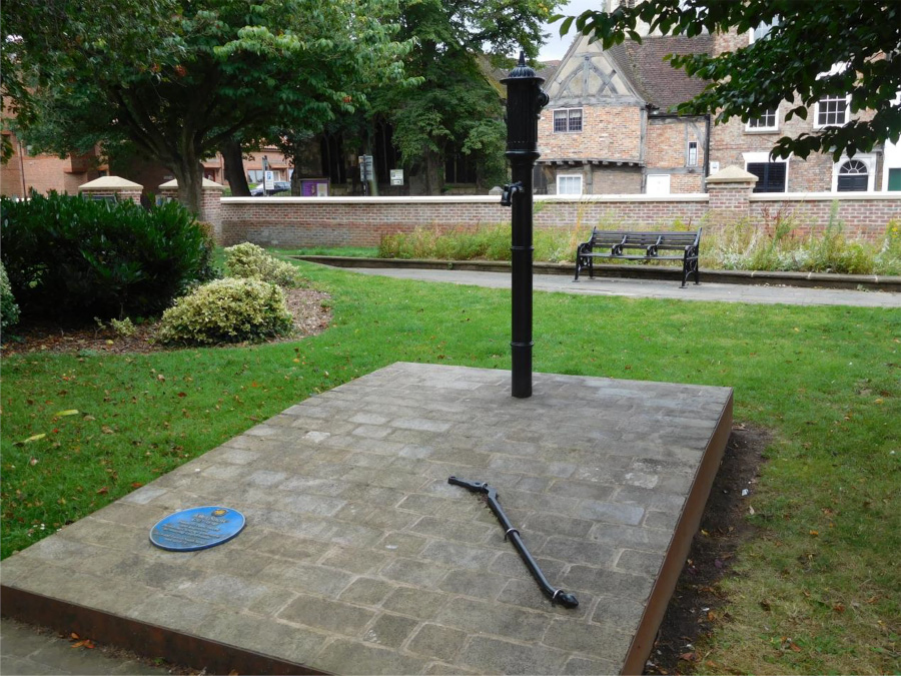
John Snow Memorial in York, erected in 2017 - water pump with handle removed, lying nearby on the ground.

Snow initially served a medical apprenticeship to a surgeon in Newcastle upon Tyne, where he also encountered a cholera epidemic for the first time, in a nearby pit village. He subsequently completed his medical training in London from 1837, gaining qualifications from the Royal College of Surgeons and the Society of Apothecaries, before earning his MD from the University of London in 1844.

His best-known memorial reflects his achievement in persuading the authorities to remove the handle from the Broad Street water pump, which he had identified as the source of the cholera outbreak in Soho, in 1854. He used his skill in epidemiology to combine several methods of determining the source and illustrated his findings, plotting the cases on a ‘dot’ map, to the Cholera Inquiry.

He was cynical about the ‘miasma theory’ which stated that disease was caused by pollution or a noxious form of ‘bad air’ and was widely believed at the time. He had already published a short pamphlet suggesting that water was the route of cholera dissemination but this had largely been dismissed. However, his lobbying resulted in the water pump handle being removed, quickly bringing the epidemic under control. Interestingly, despite his recommendations reducing the threat, his theory of faecal–oral transmission was not widely believed.

He updated his publication into a second edition in 1855 ‘On the Mode of Communication of Cholera’. However, less than 100 copies were sold and he was the subject of much criticism, including contempt from the Editor of The Lancet at the time and others. One of his main critics was Edmund Parkes, an army physician with much experience in ‘military hygiene’ (public health). He argued that the geographical distribution of cases around the Broad Street pump was totally consistent with miasmatic, airborne transmission and he found Snow's evidence for waterborne transmission to be unconvincing.^8–10^ With the benefit of hindsight and advances in science, we know, of course, that Snow was correct and we now see what a pioneer he was in this field – it was only after his death that his theories on the transmission of cholera were finally accepted.^11^

The replica water pump, without handle, originally installed in 1992, was removed in 2015 for local area development and refitted in its original location in July 2018, in Broadwick Street, Soho^12^ (Figure 2). There is also a nearby National Chemical Landmark blue plaque, erected by the Royal Society of Chemistry in 2008.^13^

**Figure 2.**
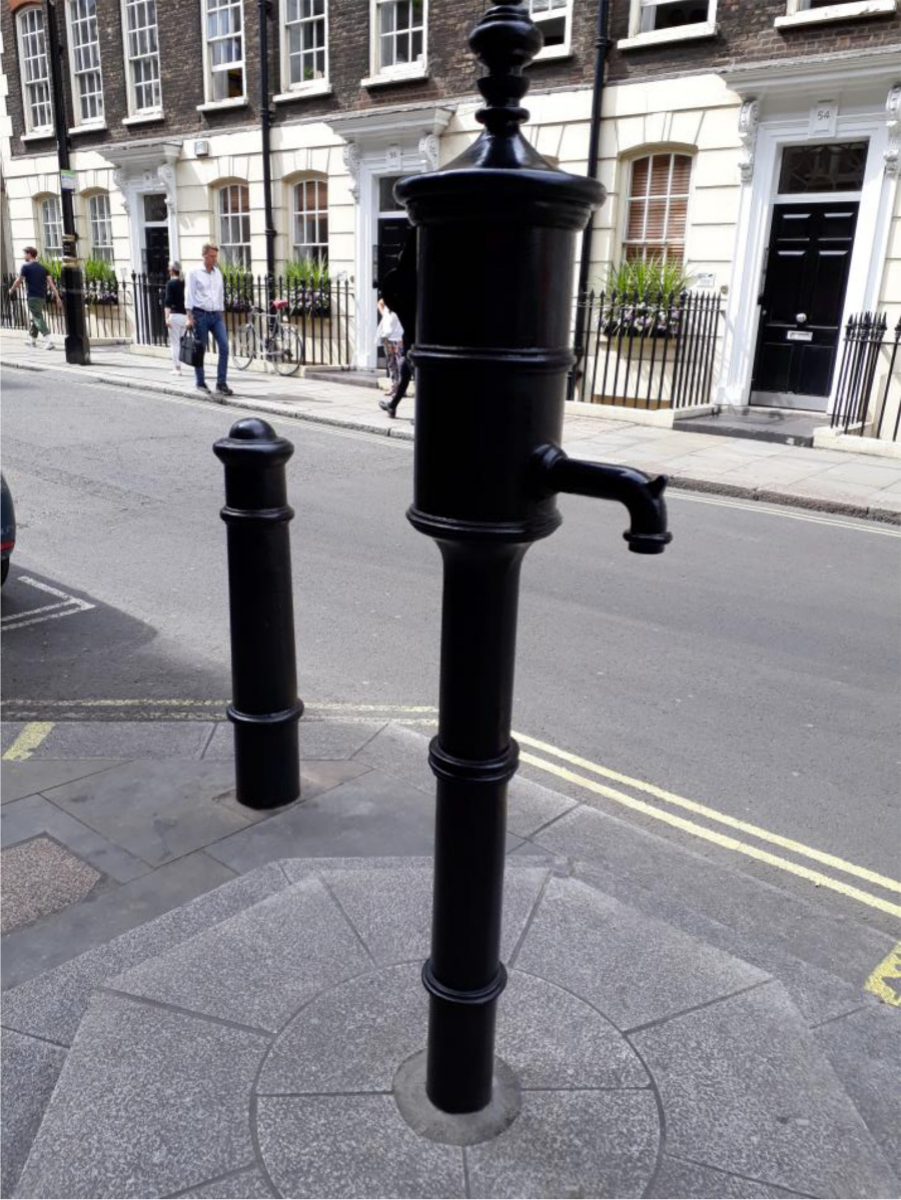
Replica water pump in Soho.

These memorials stand adjacent to the eponymous John Snow pub (Figure 3), which is well worth a visit; the first floor features many pictures and items of interest about Snow and the Broad Street pump. Also, membership of the John Snow Society has only one requirement – which is to visit this pub on any trip to London! There seems a sense of irony that someone who was teetotal for most of his life has an eponymous pub.

**Figure 3.**
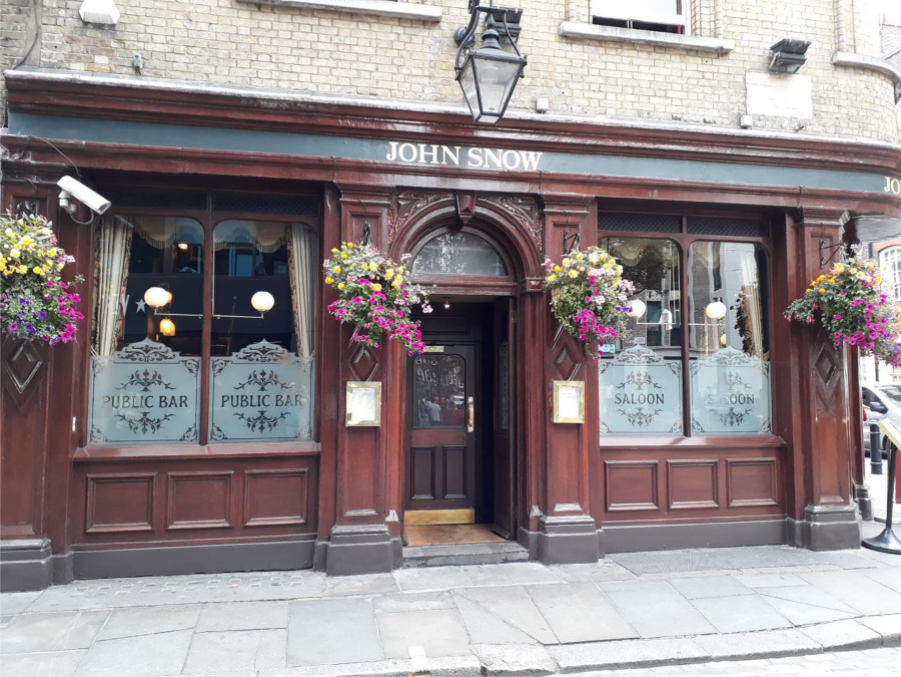
The John Snow Public House in Soho.

Snow is equally celebrated for his research into and practice in anaesthesia.^1,14^ He experimented with ether, bringing his laboratory work and clinical experience together. He designed equipment to mix an accurate dose of ether with air and understood the dependence of the mixture on temperature.^3^

Chloroform had been introduced in the mid-1800s and Snow applied the same scientific rigour to the development of chloroform administration, which is more potent than ether. He recommended that a vaporiser should be used to improve safety, allowing for accurate, controlled delivery.

Originally introduced for obstetric anaesthesia by James Young Simpson, chloroform did not gain initial popularity due to much opposition from both the medical profession and the church. In the mid-1850s, interfering with the ‘natural’ process of childbirth was seen as unhelpful, at best and frankly unethical, at worst.

However, Snow established a safer practice using chloroform in labour, by delaying its use until the second stage of labour and also limiting the dose. He achieved the feat of obtaining satisfactory analgesia with a safer technique. Queen Victoria asked Snow to administer chloroform to her, during the delivery of the last two of her nine children – Prince Leopold in 1853 and Princess Beatrice in 1857. Medical and religious acceptance soon followed and this has been largely attributed to Queen Victoria's experiences. This may actually be too simplistic a view since it is possible that Snow's medical colleagues were already changing their views on the use of chloroform as they were aware of his successful obstetric practice.^14^

Above the door at John Snow's second London house at 54 Frith Street is a blue plaque, commemorating his illustrious career. The plaque was created and placed by the Association of Anaesthetists of Great Britain and Ireland in the early 1980s.^15^ There had been some dispute over the exact location of his house, but a detailed investigation of street numbering at the time showed it to be correct.^16^

He died from a stroke aged 45, in 1858. He was buried in London's Brompton Cemetery, where his final memorial lies.

Snow's pioneering work in medicine, anaesthesia and epidemiology is immense. The Lancet corrected their initial obituary of Snow, 200 years after his birth, recognising his remarkable achievements.^11^ Amongst many accolades, we can remember him with the benefit of hindsight, as the most accomplished anaesthetist in Britain during his lifetime^1^ and the hero of epidemiology.^2^
